# Genetic Alterations in Members of the Proteasome 26S Subunit, AAA-ATPase (*PSMC*) Gene Family in the Light of Proteasome Inhibitor Resistance in Multiple Myeloma

**DOI:** 10.3390/cancers15020532

**Published:** 2023-01-15

**Authors:** Larissa Haertle, Natalia Buenache, Hipólito Nicolás Cuesta Hernández, Michal Simicek, Renata Snaurova, Inmaculada Rapado, Nerea Martinez, Nieves López-Muñoz, José María Sánchez-Pina, Umair Munawar, Seungbin Han, Yanira Ruiz-Heredia, Rafael Colmenares, Miguel Gallardo, Margarita Sanchez-Beato, Miguel Angel Piris, Mehmet Kemal Samur, Nikhil C. Munshi, Rosa Ayala, Klaus Martin Kortüm, Santiago Barrio, Joaquín Martínez-López

**Affiliations:** 1 Department of Hematology, Hospital Universitario 12 de Octubre, Spanish National Cancer Research Center (CNIO), Complutense University Madrid, 28041 Madrid, Spain; 2 Department of Internal Medicine II, University Hospital Würzburg, 97080 Würzburg, Germany; 3 Kinases, Protein Phosphorylation and Cancer, Structural Biology Program, Spanish National Cancer Research Centre (CNIO), 28029 Madrid, Spain; 4 Haematology, Ostrava University Hospital, 70300 Ostrava, Czech Republic; 5 Faculty of Medizine, Ostrava University, 70300 Ostrava, Czech Republic; 6 Centro de Investigación Biomédica en Red (CIBER) de Cáncer (CIBERONC), 28029 Madrid, Spain; 7 Departamento Hematopatología Translacional, IDIVAL, Instituto de Investigación Marqués de Valdecilla, 39011 Santander, Spain; 8 Altum Sequencing Co., 28005 Madrid, Spain; 9 Medical Oncology Department, Lymphoma Research Group, Hospital Universitario Puerta de Hierro-Majadahonda, IDIPHISA, 28220 Madrid, Spain; 10 Pathology Department, Instituto de Investigación Sanitaria Fundación Jiménez Díaz, 28040 Madrid, Spain; 11 Department of Data Science, Dana Farber Cancer Institute, Boston, MA 02215, USA; 12 Department of Biostatistics, Harvard T.H. Chan School of Public Health, Boston, MA 02215, USA; 13 Jerome Lipper Multiple Myeloma Center, Department of Medical Oncology, Dana Farber Cancer Institute, Boston, MA 02115, USA; 14 VA Boston Healthcare System, Boston, MA 02115, USA

**Keywords:** Multiple Myeloma, drug resistance, proteasome inhibitors, *PSMC2*, immunoglobulin rearrangement, ATPase activity

## Abstract

**Simple Summary:**

In comparison to other neoplasias, Multiple Myeloma is extremely sensitive to changes in protein homeostasis. Therefore, proteasome inhibitors are highly efficient and widely used for this disease. Genetic alterations of the *PSMC* genes, such as the ones shown in this manuscript, affect this Multiple Myeloma vulnerability and, therefore, play a key role in the physiopathogenesis and resistance to proteasome inhibitors. By different modes of action, those alterations are likely to increase the proteolytic capacity of cancer cells. Even though they occur in low frequencies, they can serve as biomarkers to predict and monitor a patient’s response to proteasome inhibitors over time and might be useful to guide therapy.

**Abstract:**

For the treatment of Multiple Myeloma, proteasome inhibitors are highly efficient and widely used, but resistance is a major obstacle to successful therapy. Several underlying mechanisms have been proposed but were only reported for a minority of resistant patients. The proteasome is a large and complex machinery. Here, we focus on the AAA ATPases of the 19S proteasome regulator (*PSMC1-6*) and their implication in PI resistance. As an example of cancer evolution and the acquisition of resistance, we conducted an in-depth analysis of an index patient by applying FISH, WES, and immunoglobulin-rearrangement sequencing in serial samples, starting from MGUS to newly diagnosed Multiple Myeloma to a PI-resistant relapse. The WES analysis uncovered an acquired PSMC2 Y429S mutation at the relapse after intensive bortezomib-containing therapy, which was functionally confirmed to mediate PI resistance. A meta-analysis comprising 1499 newly diagnosed and 447 progressed patients revealed a total of 36 SNVs over all six *PSMC* genes that were structurally accumulated in regulatory sites for activity such as the ADP/ATP binding pocket. Other alterations impact the interaction between different PSMC subunits or the intrinsic conformation of an individual subunit, consequently affecting the folding and function of the complex. Interestingly, several mutations were clustered in the central channel of the ATPase ring, where the unfolded substrates enter the 20S core. Our results indicate that *PSMC* SNVs play a role in PI resistance in MM.

## 1. Introduction

The 26S proteasome is a large multicatalytic molecular machinery for protein degradation. It is composed of at least 33 distinct proteasomal subunits [1] that can be differentiated into two parts, the 19S regulatory complex and the 20S catalytic core [2]. The 19S complex itself is further composed of the 19S lid (encoding genes are named *PSMD*s) and the 19S hexameric AAA (ATPases are associated with diverse cellular activities) proteins (encoded by the *PSMC* genes). The *PSMD* subunits are essential for recognizing ubiquitinated substrates and orchestrating their degradation [3]. The *PSMC*s unfold and introduce the substrates into the catalytic 20S core [4], encoded by the *PSMA* and *PSMB* genes and composed of two outer α rings and two inner β rings [5,6]. Proper proteasome function for the basal protein turnover and degradation of unfolded proteins is essential to all cells. Multiple myeloma (MM) cells produce excessive amounts of immunoglobulins, making them highly dependent on proteasome function [7]. Proteasome inhibition leads to the accumulation of misfolded and unfolded proteins in the cell, which induces proteotoxic stress and ultimately apoptosis [8]. Therefore, in MM, proteasome inhibitors (PIs) such as bortezomib (BTZ), carfilzomib (CFZ), and ixazomib (IXA) are highly efficient and widely used [9,10]. New PIs such as oprozomib, delanzomib, and marizomib are currently under clinical investigation [2]. All clinically available PIs for MM therapy covalently or reversibly target the β5 subunit of the proteasome, which is part of the 20S catalytic core and encoded by *PSMB5* [2,11]. The PIs have substantially enhanced the treatment of MM in the past two decades [9,12]. However, MM remains incurable, and most patients inevitably develop drug resistance [13,14]. Patients can be directly unresponsive to first-line therapy, or they can primarily respond and acquire resistance later on under treatment when the disease progresses. The underlying resistance mechanisms are manifold and affect different modes of gene regulation. For example, genomic mutations in *PSMB5*, which induce steric or conformational changes in the proteasome drug-binding site [11] or the downregulation of single subunits of the 19S regulatory complex that enhance the proteolytic capacity of the cell [15] inter alia by epigenetic dysregulation of *PSMD5* [16]. Several further 19S subunits, e.g., *PSMD2*, *PSMD12*, *PSMC5*, and *PSMC6* [15,17,18,19], and other gene networks have also been associated with PI resistance [20,21,22]. Generally, resistance is supposedly the product of genome instability, clonal diversity, tiding, evolutionary cancer adaptation, and MM´s unique relationship with the bone marrow microenvironment [23]. Still, to date, the described modes of action only apply to a minority of patients, and the interactions of the proteostasis network are not fully understood. 

The *PSMC* AAA ATPase gene family, which comprises six members (*PSMC1* to *PSMC6*), forms a heterohexameric ring that flanks the entry port of the 20S proteasome. Folded ubiquitinated proteins require the AAA-ATPases to unveil an unstructured shape and to open the α ring channel of the 20S core particle [24]. Cycles of ATP binding and hydrolysis become translated into mechanical force, inducing the conformational change in the unfolding process, and the AAA-ATPases pull the polypeptide chain into the proteolytic cavity, creating a peristaltic movement [24].

In this manuscript, we start with the identification of a *PSMC2* mutation acquired under intense PI treatment in an index patient and a functional confirmation that this mutation induces drug resistance in vitro. Then, we expand towards a generic meta-analysis of *PSMC* mutations and predict their structural impact on the proteasome, based on 3D modeling.

## 2. Material and Methods 

### 2.1. Characteristics of the Index Patient

**Figure 1:**
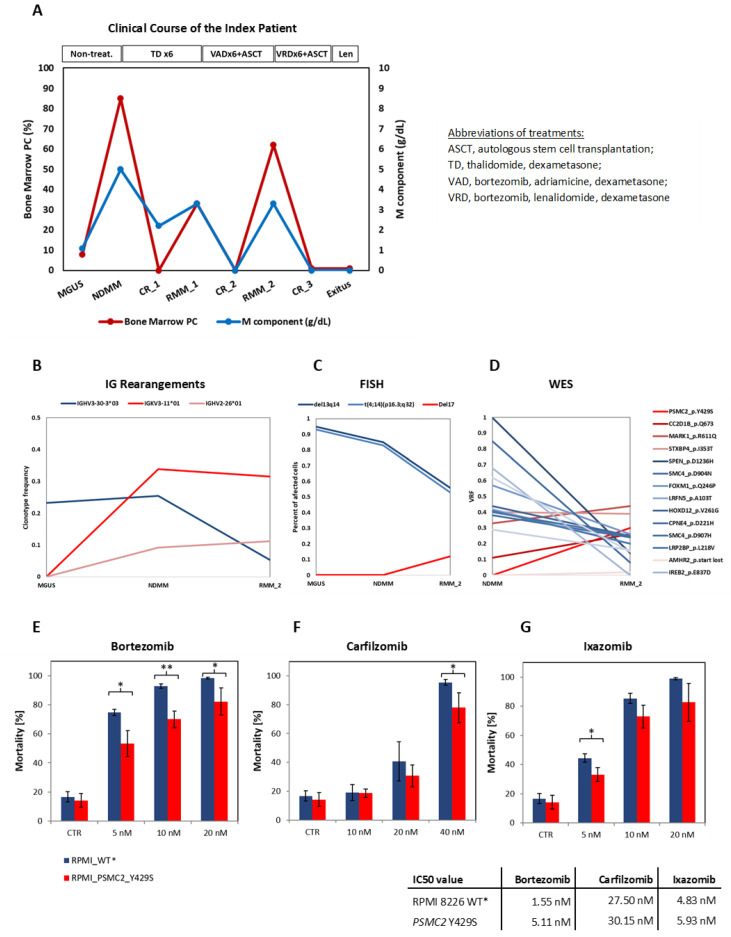
Deep analysis of the index patient in longitudinal samples: clinical course, treatment regimen, and sampling time points (**A**); oligoclonality by immunoglobulin sequencing (**B**); FISH (**C**); WES (**D**) and functional confirmation of the PSMC2 p.Y429S mutation (**E**–**G**). Cytotoxicity assay and IC50 values of the RPMI 8226 MM cell line harboring stably expressed PSMC2 p.Y429S mutation and the control (* RPMI 8226 transduced with the lentivirally encoded WT PSMC2).

The clinical course and treatment of the index patient is presented in Figure 1A. This patient died due to pneumococcal pneumonia, when he was in complete response (CR). Before, he suffered from two relapses. Sequential samples were collected for different time points, starting with monoclonal gammopathy of undetermined significance (MGUS), over newly diagnosed myeloma (NDMM) to relapse after intensive PI-containing therapy (RMM_2). These three time points were analyzed for immunoglobulin (IG) rearrangements and fluorescent in situ hybridization (FISH). Whole exome sequencing (WES) data were available only for NDMM and RMM_2. 

### 2.2. Clonal Immunoglobulin (IG) Quantification

In this study, an immunoglobulin deep-sequencing method for identifying and quantifying Minimal Residual Disease (MRD) was applied, as previously described in Martinez-Lopez et al. in 2017 [25] and patented in Barrio et al. in 2017 (European patent EP3018214A1). To amplify all IGH or IGK sequences, this method uses standardized primers developed by the Biomed-2 consortium [26]. Sequencing was performed on the Ion Proton sequencing platform (ThermoFisher Scientific, Waltham, MA, USA). 

### 2.3. Fluorescent In Situ Hybridization (FISH)

Systematic screening for genomic aberrations via interphase FISH was conducted as previously described [27]. The European Myeloma Network FISH workshop recommendation [28] was followed for the definition of a cutoff level of 10% for translocations (fusion/break-apart probes).

### 2.4. Whole Exome Sequencing (WES)

WES was performed at Centro Nacional de Análisis Genómico (CNAG) in Barcelona, Spain, following standard protocols for high-throughput paired-end 76bp sequencing on the Illumina HiSeq2000 platform (Illumina Inc., San Diego, CA, USA). The variant calling was conducted using a calling software written in-house for potential mutations showing minimum independent multi-aligner evidence. The mean coverage of the two serial samples and the germline was 93x, and a mean of 85% of bases were covered with at least 20x.

### 2.5. Exome Sequencing Validation

To validate the results obtained by exome sequencing, a targeted deep sequencing panel against the regions of interest was designed. Ion AmpliSeq Designer (ThermoFisher Scientific) was used to design customized oligos for multiplex PCR amplification of 200 bp amplicons. Libraries were prepared with the IonOneTouch2 and IonOneTouch ES automated systems (ThermoFisher Scientific). Sequencing was conducted using the 318^TM^ chip and semiconductor-sequencing technology (IonTorrent PGM, ThermoFisher Scientific), in accordance with the instructions of the manufacturer [29]. Raw data were aligned and indexed as BAM and BAI files using the Torrent Suite software, and variants were called with the Torrent Variant Caller and annotated using the Ion Reporter software (ThermoFisher Scientific). We used PolyPhen-2 [30], and MutationTaster2 [31] to assess the impact of mutations on protein function. The serial samples were sequenced with an average coverage of 12.000x (10–400.000x). The allele frequency (AF) was corrected by the purity of the sample defined by the relative number of CD138+ plasma cells, which was 84% for the NDMM and 64% for the RMM sample. Variants with AF less than 1% or with less than 20x coverage were considered non-validated. 

### 2.6. Functional Validation of the PSMC2 Y429S Point Mutation

Via site-directed mutagenesis, a lentiviral vector system harboring the *PSMC2* g.chr.7:103008485:A>C p.Y429S mutation was generated. Lentivirus expressing WT-PSMC2 and mutant-PSMC2 Y429S was used to infect RPMI 8226 cells to overexpress the WT or mutant protein, respectively. Selection of the infected cells was conducted with puromycin (24 h). After an incubation time of 48 h with different doses of the PIs BTZ, CFZ, and IXA, the cell viability was measured using 3-(4,5-dimethylthiazol-2-yl)-2,5-diphenyltetrazolium bromide (MTT) compound and CellTiter 96^®^ AQueous One Solution (Promega, Madison, WI, USA). The measurement was performed in triplicates. The GraphPad Prism software v.8 (La Jolla, CA, USA) was used to determine the IC50 value by a nonlinear regression model.

### 2.7. Meta-Analysis of WGS/WES Cohorts

We analyzed WGS data from MM patients treated in the University Hospital of Würzburg (N = 130 samples) that was published in Haertle et al. [16] and partly also published in Truger et al. [32], CoMMpass (WES, IA17 release, https://themmrf.org, accessed on 15 January 2022), and other published [33,34,35,36] datasets. The WGS dataset from the Dana–Farber Cancer Institute, Boston (N = 362 NDMM patients), was partly published (N = 183 NDMM patients) [37] and partly unpublished (N = 179 NDMM patients). In total, the meta-analysis comprises 1946 MM cases. Of these, 1499 of the patients were NDMM and 447 were pretreated MM (PMM). We analyzed the incidence of single nucleotide variants (SNVs) and small deletions (≤50 bp) in the six proteasomal *PSMC* genes, *PSMC1*, *PSMC2*, *PSMC3*, *PSMC4*, *PSMC5*, and *PSMC6* (Supplementary Tables S2 and S3), and located them in the whole complex (Supplementary Figure S1).

### 2.8. Structural Analysis

The structural analysis was performed with the University of California, San Francisco (UCSF)’s ChimeraX software [38] and data from public databases (Protein Data Bank) PDB 5GJR [39] (Supplementary Figure S1). Further 3D modeling was conducted with PyMol Molecular Graphics System (DeLano Scientific) using PDB 5GJQ [39].

## 3. Results

### 3.1. Clinical Course of the Patient

The patient was diagnosed with IGAk MGUS and progressed three years later to symptomatic myeloma. Then, he was treated with a thalidomide-based regimen, achieved complete response (CR_1), and progressed (RMM_1) (Figure 1A). Next, he was treated with a bortezomib-based regimen (VAD) and ASCT achieving CR_2. Nine months after that, the patient progressed again (RMM_2) and was treated with VRD and ASCT. The patient died of acute pneumococcal pneumonia when he was in CR_3 under lenalidomide maintenance therapy.

### 3.2. Identification of Clonal Diversity in the Index Patient Using IG Rearrangement Quantification, FISH, and WES 

To study the clonal characteristics of the index patient, we applied an in-house method to quantify clonal IG rearrangements by NGS using Biomed II primers. Based on the clonal dynamic analysis, at least two different clones were identified (Figure 1B). Clone one (IGHV3-30-3*03) was the only one detected in MGUS, with a clonotype frequency (CF) of 23.3%. 

It was also present at NDMM (25.5%) and decreased, but it was still detectable at the second relapse (5.3%). Clone two (IGKV3-11*01 and IGHV2-26*01) was absent in MGUS but was predominant in NDMM with 39.9% and 9.2%, respectively, and in RMM_2 with 31.6% and 11.2%, respectively. Notably, the two clones were detected at MRD levels in the CR samples collected before and after the first relapse (Supplementary Figure S3).

On the other hand, the FISH analysis also identified two major clones (Figure 1C). The first one, dominant in MGUS and NDMM, was characterized by the t(4;14)(p16.3;q32) and del13q14 (95% and 93% at MGUS and 85% and 83% at NDMM). At the second relapse, these chromosomal alterations were only detected in 56% and 53% of the CD138+ cells, respectively, but a new independent clone harboring del17p emerged in 12% of the cells. The NDMM and RMM_2 samples had enough material left to further perform a WES analysis (Figure 1D). In total, 58 variants were identified, of which 51 were validated by targeted sequencing. Nine variants were excluded from the analysis as they were located in IG genes. Another 26 variants were located in intronic regions or annotated as coding synonymous by the PolyPhen-2 database. The remaining 16 variants were annotated as somatic mutations, as previously described in cancer patients, and are listed in Supplementary Table S1. Comparing the NDMM and the RMM samples, 4/16 mutations showed an increased VAF in RMM_2, whereas the other 12 showed a VAF reduction between the two time points (Figure 1D). Interestingly, the only mutation that was entirely absent in NDMM and only acquired in RMM_2 with a VAF of 30% was PSMC2 Y429S. As this mutation affects the 26S proteasome AAA ATPase 2, and the sampling was conducted after six cycles of VAD therapy, we further functionally explored its role in PI resistance.

### 3.3. The PSMC2 Y429S Single Nucleotide Variant Mediates PI Resistance In Vitro

The relevance of the *PSMC2* g.chr.7:103008485:A>C (Y429S) mutation in PI resistance was functionally confirmed by comparing the PI response of the RPMI 8226 cell line expressing PSMC2^Y429S^ versus PSMC2^wt^. In the cytotoxicity assay, the mutant counterpart showed significantly increased resistance to BTZ at all applied concentrations: 5 nM (*p* = 0.015), 10 nM (*p* = 0.0031), and 20 nM (*p* = 0.0402) (Figure 1E). For CFZ, statistical significance was only reached at 40 nM (*p* = 0.0449) (Figure 1F) and, for Ixazomib, it was only reached at 5 nM (*p* = 0.0229) (Figure 1G).

### 3.4. Incidence of Genomic Alterations in PSMC Family Member Genes

**Figure 2:**
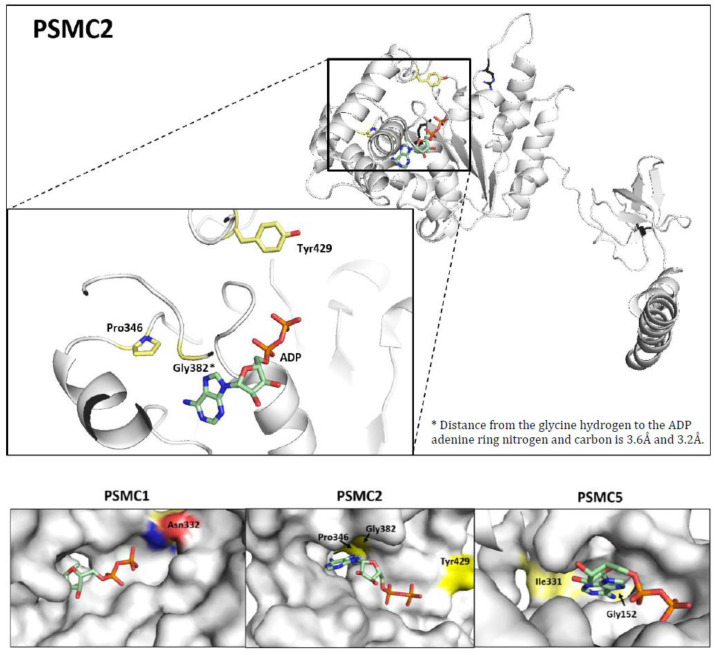
Amino acids with patient-derived mutations in proximity to the ATP/ADP pocket. PDB 5GJQ [39] analyzed with PyMol. The ADP is shown as wireframe stick in light green, and the endogenous amino acids where the patient-derived mutations were found are shown in light yellow, with red indicating a negative charge (oxygen) and blue indicating a positive charge (nitrogen).

The meta-analysis identified 34 MM patients with SNVs in *PSMC* genes (Supplementary Table S2, Supplementary Figure S1). Of them, 27 SNVs were identified within the NDMM cohort (27/1499, mutation incidence: 1.80%) and 7 in the PMM cohort (7/447, mutation incidence: 1.57%) (Supplementary Table S3). With 0.67% (10/1499), *PSMC5* was the gene with the highest mutation rate in NDMM, while it was *PSMC6,* with 0.67% (3/447), in PMM. One patient from CoMMpass exhibited three independent SNVs, all of them within the *PSMC5* gene (D299H, K402N, and W405L) (Supplementary Table S2). Interestingly, the PSMC5 G152S mutation was found in three independent patients, and a G152R was found in a fourth patient. PSMC5 G152, therefore, represents a potential mutational hotspot (Supplementary Table S2). Furthermore, PSMC5 Gly152 is located within the ADP/ATP binding pocket and is in direct interaction with adenine (Figure 2 and Figure S2). Of note, the mutation incidence of PSMC2 slightly increased from 0.27% in NDMM to 0.45% in PMM.

### 3.5. Prediction of the Structural Impact of the Mutations

Based on observations on the WT 26S proteasome complex (PDB 5GJQ and PDB 5GJR [39]), the PI-resistance mutation PSMC2 Y429S was topologically identified as being near the positive pockets formed by the other protomers of the 26S complex and the theoretical place of the gamma phosphate of the ATP. Other mutations identified by the meta-analysis also cluster in the ADP/ATP pockets (Figure 2). Several of them affect a glycine-rich loop, including four patients with the same mutated G152(R/S) amino acid. (Figure 2 and Figure S2). Of note, this glycine-rich loop is comparable to the P loop known from other protein kinases [40]. Other groups of mutations were identified affecting PSMC protomer interactions or impairing the intrinsic conformation of the respective PSMC subunits (Supplementary Figure S4).

## 4. Discussion

In this study, we show, in an exemplary patient, that the clonal evolution behind a relapse can be made visible by integrating different genomic approaches and comparing serial samples. Furthermore, our meta-analysis of NDMM and progressed MM patients reveals that, even though *PSMC* SNVs occur in low frequencies, they are likely to confer PI resistance.

**Figure 3:**
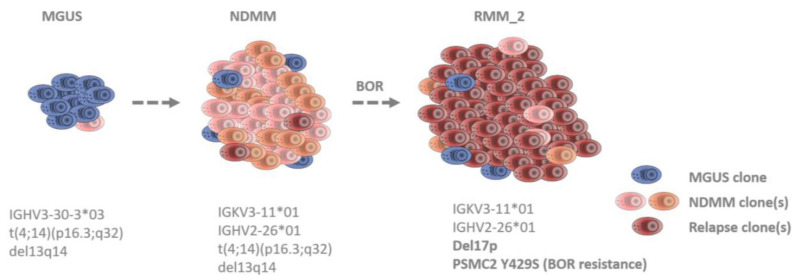
Clonal evolution in the index patients at different disease time points. The deep sequencing analysis of the index patient reveals that the tumor was composed of and dominated by different MM clones at the different time points: MGUS, NDMM, and RMM_2. At least three independent MM clones were confirmed by the IG rearrangement analysis, FISH, and WES.

MM is known to be a clonally heterogeneous cancer [41]. At the clinical level, current treatment regimens are able to widely control MM. Nevertheless, a patient’s cure begins to become uncertain when tumor cells persist through therapy. Drug-resistant clones and subclones can already be present at diagnosis, or mutations and/or epimutations can be newly acquired, e.g., under the selective pressure of the treatment [42,43,44,45,46]. Which clone becomes dominant at a time point depends on the clone-specific properties as well as on the surrounding environment. The analysis of our index patient reveals the existence of at least three independent MM clones in the course of the disease from MGUS, over NDMM, to the second PI-resistant relapse (Figure 3). The first clone was already present at MGUS and is characterized by IGHV3-30-3*03 (Figure 1B). At the same time point, FISH also identified a translocation (4;14)(p16.3;q32) and a del13q14 (Figure 1C). The second clone was absent at MGUS and arose at NDMM. Consequently, this clone must be responsible for the progression. It is characterized by two different IG rearrangements, IGKV3-11*01 and IGHV2-26*01 (Figure 1B). IG oligoclonality from MGUS to MM has been described previously as a phenomenon that, in some cases, can occur due to unrelated sequences (which refer to independent clones and, thus, independent myelomas) [47]. At the second relapse, all three IG rearrangements were still detectable; thus, both clones were still present. However, in the FISH, a newly acquired del17p appeared (Figure 1C) and is, therefore, representative of the existence of a third clone or sub-clone. Del17p is considered a high-risk mutation, conferring extra proliferative fitness to the tumor [48]. Moreover, at this time point, an acquired drug-resistance mutation (PSMC2 Y429S) was identified in the WES analysis (Figure 1D). This mutation was functionally validated to mediate PI resistance in vitro (Figure 1E). Thus, clone three must be responsible for the relapse.

Our meta-analysis indicates that PSMC mutations accumulate in important regions for the ATPase function, e.g., the ADP/ATP binding pocket (Figure 2) or the central core of the ATPase complex, where the substrates become introduced into the catalytic 20S complex (Supplementary Figure S4). We also found SNVs affecting the conformation of either one individual PSMC subunit or the protomer interactions of several subunits (Supplementary Figure S4). Those SNVs are likely to impact the 3D structure of the ATPase complex as a whole. Although the meta-analysis remains descriptive, and predicting the biological impact based on 3D structural modeling is speculative, there is further evidence in the literature underpinning the role of *PSMC* gene alterations in PI resistance. For instance, a CRISPR genome-wide screening identified *PSMC6* to confer BTZ resistance. An individual depletion of all other PSMC subunits by sgRNA also resulted in BTZ resistance in the MM cell lines RPMI 8226 and KMS11 [19]. Another group performed a screen applying MG132 or BTZ to KBM-7 cells and identified *PSMC2*, *PSMC3*, *PSMC4*, *PSMC5,* and *PSMC6* as key genes of PI resistance. A shRNA-mediated downregulation of PSMC5 increased PI resistance [17]. Additionally, *PSMC6* was further listed in a CRISPR-Cas9 screen performed by Sheffer et al. [49], and *PSMC1* and *PSMC6* were came up in a next-generation shRNA library screening by Acosta-Alvear et al. [18]. In MM patients, reduced 19S subunit levels at baseline correlated with an inferior disease (PFS) under BTZ treatment. In these patients, BTZ therapy was not superior to dexamethasone treatment in the controls [15]. Similarly, in patients that underwent CFZ-based combination therapy, intracellular flow cytometry of the CD138+ primary MM cells revealed that complete responders had significantly higher PSMC2 protein levels compared to partial responders [18]. PI resistance can be mediated in different ways: by increasing the proteasomal activity or the quantity of 26S proteasome present in the cell, by the regulation of other pathway proteins (e.g., autophagy), or by influencing the binding of PIs to PSMB5, just to name some. This argues in favor of the existence of many different resistance mechanisms, which are only present in a subgroup of PI-resistant patients. The integration of confirmed PI-resistance biomarkers and the identification of new ones would allow for not only the retrospective understanding of a relapse but also the prediction of a potential relapse before it occurs and, moreover, an intervention, e.g., by changing the treatment regiments once a resistant clone got detected. The number of molecular drug-resistance biomarkers is constantly rising, and an enlargement of the mechanistic understanding is equally important as the improvement of early detection methods.

## 5. Conclusions

In conclusion, this study unravels the clonal evolution and the rise of a PI-resistant mutation in an index patient. It also lists several patient-derived *PSMC* SNVs derived from a meta-analysis, with a potential link to PI resistance in MM. We give a biological rationale for further studies on exploring the exact underlying mechanisms as well as their potential as biomarkers for predicting and monitoring PI response.

## Data Availability

All requests for raw and analyzed data and materials will be promptly reviewed by the Hospital Universitario 12 de Octubre to determine whether they are subject to any confidentiality or data protection obligations. Any data and materials that can be shared will be released via a Material Transfer Agreement. A complete whole-exome raw dataset for a single patient cannot be shared according to European law. Processed data, from which the identification of a patient is not possible, can be made available. For requests, contact S.R. via e-mail at santibarrio0.3@gmail.com.

## Supplementary Materials

The following supporting information can be downloaded at: https://www.mdpi.com/article/10.3390/cancers15020532/s1, Table S1: Validated, somatic, non-synonymous mutations of the index patient. Table S2: *PSMC* single nucleotide variants of the meta-analysis and their potential structural impact. Table S3: Frequency of *PSMC* SNVs in different NDMM and PMM cohorts. Figure S1: Distribution of mutations in the PSMC subunits of the 19S Base AAA ATPase protomer. Figure S2: Map of the amino acids in direct interaction with the ADP shown for PSMC5. Figure S3: Follow-up of the index patient by using IG rearrangements analysis including MRD. Figure S4: Examples of amino acids that provoke intrinsic conformational changes or disturb the interactions within different protomers when mutated and zoom into the core of the PSMC complex. References [16,33,34,35,37,39] are cited in the supplementary materials.
