# Supplementary material for: Genetic Alterations in Members of the Proteasome 26S Subunit, AAA-ATPase (PSMC) Gene Family in the Light of Proteasome Inhibitor Resistance in Multiple Myeloma
Source: Cancers (Basel). 2023 Jan 15;15(2):532. doi: 10.3390/cancers15020532 (PMC9856285; doi:10.3390/cancers15020532)
Supplement: Supplementary file 1 [file cancers-15-00532-s001.zip › cancers-2106028-SI.pdf]

Supplementary Information

Supplemental Table S1: Validated, somatic, non-synonymous mutations of the index patient.

Gene	Chr.	Position	Ref. Allele	Alt. Allele	VAF at NDMM	VAF at RMM	Transcript	HGVS Coding	Variant Classification	Polyphen Prediction
SPEN	Chr1	16256441	G	C	1	0.14	NM_015001	p.D1236H	disease causing	missense
CC2D1B	Chr1	52821913	G	A	0.11	0.26	NM_032449	p.Q673*	disease causing	nonsense
MARK1	Chr1	220826538	G	A	0.33	0.44	NM_018650	p.R611Q	disease causing	missense
HOXD12	Chr2	176965457	T	G	0.44	0.25	NM_021193	p.V261G	disease causing	missense
CPNE4	Chr3	131388539	C	G	0.85	0.08	NM_140808	p.D221H	disease causing	missense
SMC4	Chr3	160146645	G	A	0.38	0.24	NM_001002800	p.D904N	disease causing	missense
SMC4	Chr3	160146654	G	C	0.4	0.25	NM_001002800	p.D907H	disease causing	missense
LRP2BP	Chr4	186294161	A	C	0.41	0.2	NM_018409	p.L218V	disease causing	missense
PSMC2	Chr7	103008485	A	C	0	0.3	NM_002803	p.Y429S	disease causing	missense
FOXM1	Chr12	2977838	T	G	0.57	0.26	NM_021953	p.Q246P	disease causing	missense
AMHR2	Chr12	53817719	A	G	0	0.02	NM_020547	p.start lost	disease causing	missense
LRFN5	Chr14	42356135	G	A	0.42	0.2	NM_152447	p.A103T	disease causing	missense
IREB2	Chr15	78786522	A	T	0.68	0	NM_004136	p.E837D	disease causing	missense
STXBP4	Chr17	53150307	T	C	0.4	0.39	NM_178509	p.I353T	disease causing	missense
MAST3	Chr19	18218419	G	A	0.29	0.16	NM_015016	p.R21Q	disease causing	missense
SAMHD1	Chr20	35526338	A	C	0.62	0.15	NM_015474	p.F545V	disease causing	missense

Abbreviations: Alt, alternative; Chr, chromosome; HGVS, Human Genome Variation Society; NDMM, newly diagnosed multiple myeloma; Ref, reference; RMM, relapsed multiple myeloma; VAF, variant allele fraction.

Supplemental Table S2: *PSMC* single nucleotide variants in the following published datasets: Samur et al. [37]; Haertle et al. [16]; Bolli et al. [34], Lohr et al. [33], CoMMpass and Giesen et al. [35] and their potential structural impact (PDB 5GJQ).

Disease state	Cohort	Gene	NCBI-Build	Chr.	Position	Variant Classification	Ref. Allele	Alt. Allele	Patient ID	HGVS coding	HGVS protein	Structural Prediction
NDMM	Samur et al.	PSMC1	GRCh38	chr14	90269508	frameshift_del	A	-	11845_D	c.995del	p.Asn332 ThrfsTer322	1
NDMM	Samur et al.	PSMC2	GRCh38	chr7	103353946	missense_var	G	T	12339	c.96G>T	p.Leu32Phe	6
NDMM	Samur et al.	PSMC5	GRCh38	chr17	63830403	missense_var	G	A	13835	c.454G>A	p.Gly152Ser	1
NDMM	Samur et al.	PSMC5	GRCh38	chr17	63829525	missense_var	G	A	901-016-B	c.128G>A	p.Arg43Gln	2
NDMM	Samur et al.	PSMC3	GRCh38	chr11	47426260	missense_var	A	T	934-002-B	c.20T>A	p.Ile7Asn	6
NDMM	Haertle et al.	PSMC1	GRCh37	chr14	90730114	missense_var	G	C	MLL_193429	c.388G>C	p.Glu130Gln	4
NDMM	Bolli et al.	PSMC2	GRCh37	chr7	103008052	missense_var	C	T	PD5883	c.1037C>T	p.Pro346Leu	1
NDMM	Bolli et al.	PSMC5	GRCh37	chr17	61907763	missense_var	G	C	PD5874	c.454G>C	p.Gly152Arg	1
NDMM	Bolli et al.	PSMC4	GRCh37	chr19	40485741	missense_var	G	T	PD5865	c.691G>T	p.Val231Leu	2
NDMM	Lohr et al.	PSMC5	GRCh36	chr17	59261495	missense_var	G	A	MM-0639-Tumor	c.454G>A	p.Gly152Ser	1
NDMM	Lohr et al.	PSMC6	GRCh36	chr14	52241739	5'flank	T	A	MM-0376-Tumor	-	-	7
NDMM	Lohr et al.	PSMC4	GRCh37	chr19	45177727	frameshift_del	A	-	MM-0606-Tumor	c.837delA	p.Thr279fs	2,3,4
NDMM	CoMMpass	PSMC5	GRCh38	chr17	63830403	missense_var	G	A	MMRF_1502	c.454G>A	p.Gly152Ser	1
NDMM	CoMMpass	PSMC4	GRCh38	chr19	39972381	missense_var	G	C	MMRF_1602	c.148G>C	p.Glu50Gln	5
NDMM	CoMMpass	PSMC1	GRCh38	chr14	90268235	missense_var	T	G	MMRF_1614	c.703T>G	p.Leu235Val	3
NDMM	CoMMpass	PSMC1	GRCh38	chr14	90263768	missense_var	C	T	MMRF_1640	c.386C>T	p.Ser129Leu	4
NDMM	CoMMpass	PSMC5	GRCh38	chr17	63829897	missense_var	C	G	MMRF_1886	c.212C>G	p.Ser71Cys	2,4
NDMM	CoMMpass	PSMC1	GRCh38	chr14	90260126	missense_var	G	C	MMRF_1965	c.69G>C	p.Lys23Asn	6
NDMM	CoMMpass	PSMC2	GRCh38	chr7	103367586	missense_var	A	C	MMRF_2245	c.1018A>C	p.Lys340Gln	5
NDMM	CoMMpass	PSMC1	GRCh38	chr14	90270213	missense_var	A	C	MMRF_2293	c.1049A>C	p.Lys350Thr	2,3
NDMM	CoMMpass	PSMC5	GRCh38	chr17	63830178	missense_var	G	A	MMRF_2300	c.310G>A	p.Asp104Asn	5
NDMM	CoMMpass	PSMC1	GRCh38	chr14	90269468	missense_var	G	A	MMRF_2595	c.953G>A	p.Gly318Glu	1
NDMM	CoMMpass	PSMC5	GRCh38	chr17	63831770	missense_var	T	C	MMRF_2626	c.1127T>C	p.Val376Ala	2
NDMM	CoMMpass	PSMC5	GRCh38	chr17	63831351	missense_var	G	C	MMRF_2699	c.895G>C	p.Asp299His	5
NDMM	CoMMpass	PSMC5	GRCh38	chr17	63831954	missense_var	G	T	MMRF_2699	c.1206G>T	p.Lys402Asn	6
NDMM	CoMMpass	PSMC5	GRCh38	chr17	63831962	missense_var	G	T	MMRF_2699	c.1214G>T	p.Trp405Leu	6
NDMM	CoMMpass	PSMC5	GRCh38	chr17	63831528	missense_var	T	C	MMRF_2714	c.992T>C	p.Ile331Thr	1

NDMM	CoMMpass	PSMC2	GRCh38	chr7	103362710	missense_var	T	G	MMRF_2913	c.447T>G	p.Ile149Met	3
NDMM	CoMMpass	PSMC4	GRCh38	chr19	39974303	missense_var	A	T	MMRF_2916	c.332A>T	p.Tyr111Phe	2,4
PMM	Haertle et al.	PSMC6	GRCh37	chr14	53173969	missense_var	A	T	MLL_165899	c.74A>T	p.Asp25Val	7
PMM	Lohr et al.	PSMC3	GRCh36	chr11	47403280	missense_var	T	G	MM-0332-Tumor	c.253A>C	p.Thr85Pro	3,4
PMM	Lohr et al.	PSMC6	GRCh36	chr14	52254707	missense_var	G	A	MM-0308-Tumor	c.602G>A	p.Ser201Asn	2,3
PMM	Giesen et al.	PSMC2	GRCh37	chr7	103008256	missense_var	G	T	H2	c.1144G>T	p.Gly382Cys	1
PMM	**Index patient**	PSMC2	GRCh37	chr7	103008485	missense_var	A	C	Santander patient	c.1286A>C	p.Tyr429Ser	2
PMM	CoMMpass	PSMC2	GRCh38	chr7	103367418	missense_var	C	T	MMRF_1179	c.850C>T	p.Arg284Cys	2,3
PMM	CoMMpass	PSMC5	GRCh38	chr17	63831420	missense_var	G	C	MMRF_1193	c.964G>C	p.Glu322Gln	5
PMM	CoMMpass	PSMC6	GRCh38	chr14	52708515	frameshift_del	AG	A	MMRF_2531	c.241delG	p.Glu81fs	7

Abbreviations: Alt, alternative; Chr, chromosome; HGVS, Human Genome Variation Society; ID, identifier; NCBI, National Center for Biotechnology Information; Ref, reference

1: mutation is located within the ADP/ATP pocket
2: mutation perturbs the interaction with other PSMC protomer(s)
3: mutation affects the intrinsic conformation of the respective subunit
4: mutation in the central core of the PSMC ATPase complex
5: mutation is not conclusive
6: mutation is located outside of the PDB 5GJQ
7: N.A.

Supplemental Table S3: Frequency of *PSMC* SNVs in different NDMM and PMM cohorts.

Samur et al. [37]; Haertle et al. [16]; Bolli et al. [34], Lohr et al. [33], CoMMpass and Giesen et al. [35].

	NDMM							PMM							
**Protea-some Subunit**	**CoMM pass**	**Bolli et al.**	**Lohr et al.**	**Haertle et al.**	**Samur et al.**	**Total Count Baseline**	**% Baseline**	**CoMM pass**	**Bolli et al.**	**Ziccheddu et al.**	**Lohr et al.**	**Giesen et al.**	**Haertle et al.**	**Total Count PMM**	**% PMM**
**n**	948	67	85	37	362	**1499**	**1499**	161	15	40	100	38	93	**447**	**447**
PSMC1	5	0	0	1	1	**7**	**0.47**	0	0	0	0	0	0	**0**	**0.00**
PSMC2	2	1	0	0	1	**4**	**0.27**	1	0	0	0	1	0	**2**	**0.45**
PSMC3	0	0	0	0	1	**1**	**0.07**	0	0	0	1	0	0	**1**	**0.22**
PSMC4	2	1	1	0	0	**4**	**0.27**	0	0	0	0	0	0	**0**	**0.00**
PSMC5	8	1	1	0	2	**10**	**0.67**	1	0	0	0	0	0	**1**	**0.22**
PSMC6	0	0	1	0	0	**1**	**0.07**	1	0	0	1	0	1	**3**	**0.67**
**Total**	17	3	3	1	5	**27**	**1.80**	3	0	0	2	1	1	**7**	**1.57**

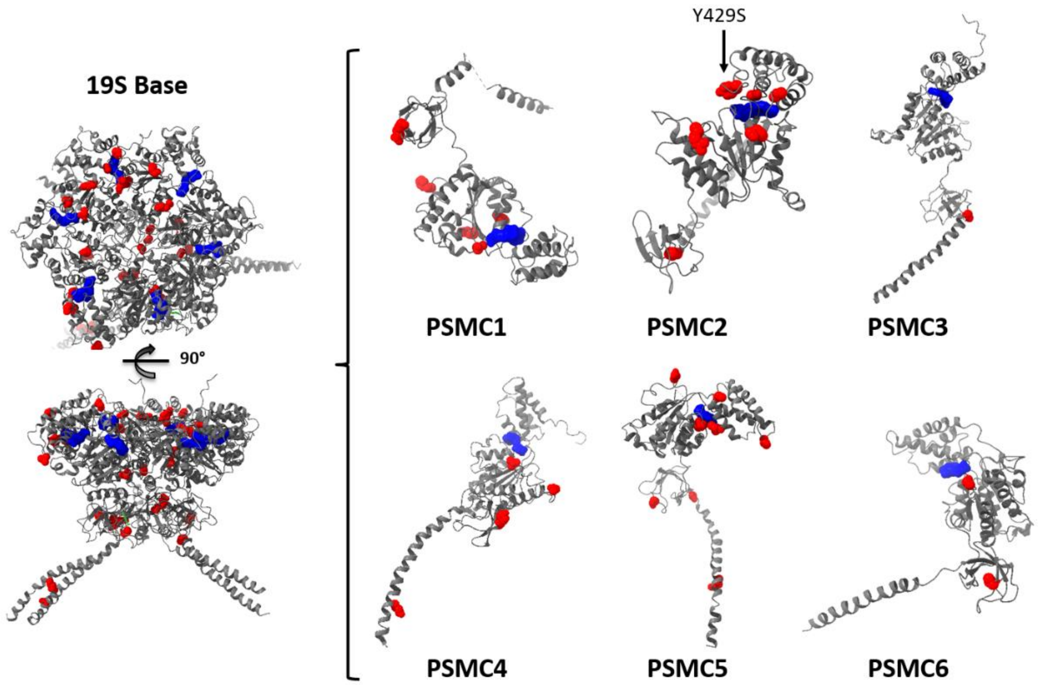

Supplemental Figure S1: Distribution of mutations in the PSMC subunits of the 19S Base AAA ATPase protomer.

Electron Microscopy (EM) structure of the 19S Base of the 26 proteasome (PDB 5GJR [39]) analyzed with the ChimeraX. The backbone of the protomer is displayed as a ribbon diagram. In the projections, the mutations are shown in red as 3D spheres and ADP in blue. Mutations cluster in proximity to the ADP/ATP binding pocket and other areas responsible for proper folding and functioning of the whole complex. The PSMC2 Y429S mutation of the index patients is indicated.

PSMC5

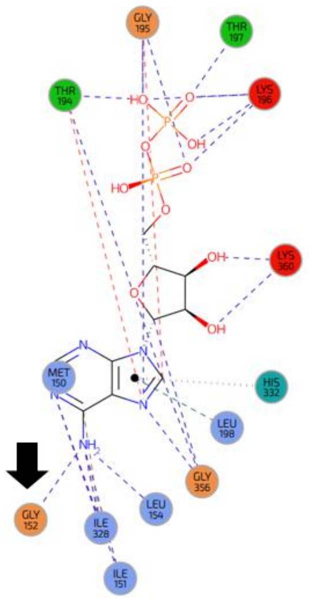

Supplemental Figure S2: Map of the amino acids in direct interaction with the ADP shown for PSMC5.
The ADP interaction maps with the different residues of the PSMC5 protomer was taken from the European Protein Data Bank (PDBE) and deposited by the authors of Huang et al. PDB 5GJQ [39]. Individual amino acids are indicated in circles; the color is related to the charge of the side chain. The black arrow highlights the PSMC5 Glycine 152, which was substituted to a serine in three independent patients and to an arginine in a fourth patient. Consequently, it might represent a mutational hotspot.

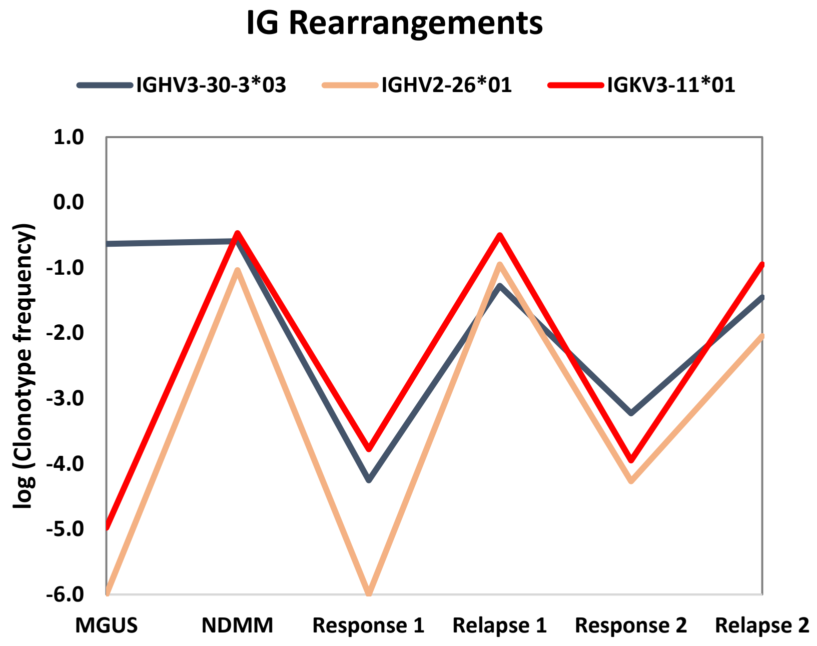

Supplemental Figure S3: Follow-up of the index patient by using IG rearrangements analysis including MRD.
In the first complete remission (response 1) IGHV3-30-3*03 and IGKV3-11*01 were detected, IGHV2-26*01 could not be detected. In the second complete remission (response 2) all 3 rearrangements were identified.

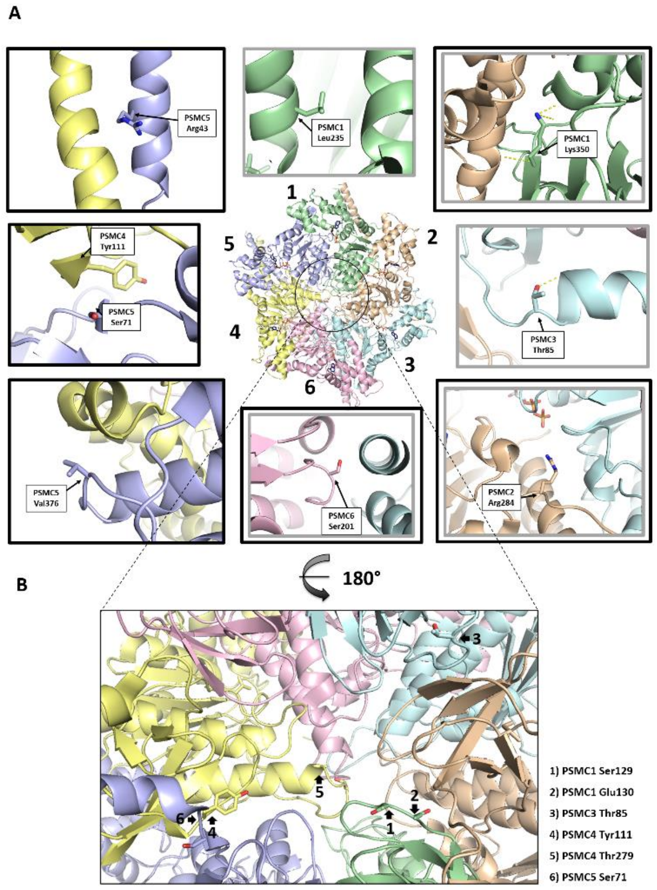

Supplemental Figure S4: Examples of amino acids that provoke intrinsic conformational changes or disturb the interactions within different protomers when mutated and zoom into the core of the PSMC complex.

(**A**) The different PSMC subunits are shown in different colors: PSMC1, 2, 3, 4, 5, and 6 in green, brown, blue, yellow, purple, and pink. The backbone is represented as a ribbon diagram, and the endogenous amino acid mutated in MM patients are shown as wireframe sticks. Those where mutations might affect the intrinsic conformation of the respective subunits are indicated by a grey frame, whereas such that might impact the proper interaction between different PSMC subunits are framed in black. (**B**) Mutated amino acids in the core of the PSMC complex through which the substrates are introduced into the catalytic 20S complex.

References

16. Haertle, L.; Barrio, S.; Munawar, U.; Han, S.; Zhou, X.; Simicek, M.; Vogt, C.; Truger, M.; Alonso Fernandez, R.; Steinhardt, M.; et al. Single Nucleotide Variants and Epimutations Induce Proteasome Inhibitor Resistance in Multiple Myeloma. Clin. Cancer Res. 2023, 29, 279 – 288.
33. Lohr, J.G.; Stojanov, P.; Carter, S.L.; Cruz-Gordillo, P.; Lawrence, M.S.; Auclair, D.; Sougnez, C.; Knoechel, B.; Gould, J.; Saksena, G.; et al. Widespread genetic heterogeneity in multiple myeloma: Implications for targeted therapy. Cancer Cell 2014, 25, 91 – 101.
34. Bolli, N.; Avet-Loiseau, H.; Wedge, D.C.; Van Loo, P.; Alexandrov, L.B.; Martincorena, I.; Dawson, K.J.; Iorio, F.; Nik-Zainal, S.; Bignell, G.R.; et al. Heterogeneity of genomic evolution and mutational profiles in multiple myeloma. Nat. Commun. 2014, 5, 2997.
35. Giesen, N.; Paramasivam, N.; Toprak, U.H.; Huebschmann, D.; Xu, J.; Uhrig, S.; Samur, M.; Bahr, S.; Frohlich, M.; Mughal, S.S.; et al. Comprehensive genomic analysis of refractory multiple myeloma reveals a complex mutational landscape associated with drug resistance and novel therapeutic vulnerabilities. Haematologica 2022, 107, 1891 – 1901.
37. Samur, M.K.; Aktas Samur, A.; Fulciniti, M.; Szalat, R.; Han, T.; Shammas, M.; Richardson, P.; Magrangeas, F.; Minvielle, S.; Corre, J.; et al. Genome-Wide Somatic Alterations in Multiple Myeloma Reveal a Superior Outcome Group. J. Clin. Oncol. 2020, 38, 3107 – 3118.
39. Leipe, D.D.; Koonin, E.V.; Aravind, L. Evolution and classification of P-loop kinases and related proteins. J. Mol. Biol. 2003, 333, 781 – 815.
